# Does Psychosocial Work Environment Factors Predict Stress and Mean Arterial Pressure in the Malaysian Industry Workers?

**DOI:** 10.1155/2018/9563714

**Published:** 2018-01-15

**Authors:** Muhammad Umair Javaid, Ahmad Shahrul Nizam Isha, Asrar Ahmed Sabir, Zulkipli Ghazali, Matthias Nübling

**Affiliations:** ^1^Department of Management & Humanities, Universiti Teknologi Petronas, 32610 Seri Iskandar, Perak, Malaysia; ^2^Freiburg Research Centre for Occupational Sciences, Freiburg, Germany

## Abstract

Psychosocial risks are considered as a burning issue in the Asia-Pacific region. The aim of this study was to investigate the impact of psychosocial work environment factors on health of petrochemical industry workers of Malaysia. In lieu to job demands-resources theory, significant positive associations were found between quantitative demands, work-family conflict, and job insecurity with stress, while a significant negative association of role clarity as a resource factor with stress was detected. We also found that quantitative demands were significantly associated with the mean arterial pressure (MAP). Multistage sampling procedure was used to collect study sample. Structural Equation Modeling was used to identify relationship between the endogenous and exogenous variables. Finally, the empirically tested psychosocial work environment model will further help in providing a better risk assessment in different industries and enterprises.

## 1. Background

History presents us with two industrial revolutions, each based on a general purpose technology. They encompassed a series of technological innovations which were small in size or scale but their impact was so powerful and far-reaching that they served as catalysts in charting the course of human progress. Steam and electricity were the driving forces behind those revolutions. The age of a third revolution is upon us, powered by computers and networks. This revolution is all set to improve people's lives in an unprecedented manner provided it is directed towards expanding the horizon of opportunities and building capabilities. By their very nature, such swift transformations are pervasive and unruly in the short term as they reform the building blocks of the society and the workplace but forbearance in the short term heralds the positive results of the long term. Consequently, some workers will be able to improvise faster and yield more positive results than their counterparts. They are in lieu to make there working life easier by handling the psychosocial work environment and reducing the efforts to subsist it.

A workplace never resides in isolation, and hence in the workplace employees experience both psychological and social conditions which often called psychosocial work environment. Psychosocial work environment has become a continuous component in studies of occupational health and stress and encompasses concerns on risks which generate from the psyche perceptions of individuals in accordance with the risks of societal environment. Over the past few decades, not only scientific researchers have considered it as an important area of inquiry, but also various national governments have emphasized and acknowledged the impact of psychosocial work environment on health, health behaviors, performance, effectiveness, and productivity of workers and organizations [1–6].

## 2. Introduction

The theme of the eleventh Malaysian plan (2016–2020) targeting vision 2020 is “*anchoring growth on people*” which visualizes it as a developed country with economic, political, spiritual, cultural, and psychological dimensions [7]. The psychological and physiological health of the workers are central to a prosperous future of Malaysian industry. However, Malaysian organizations are facing a growing work-related stress concern with 70% of the Malaysian employees affected by high work-related stress with 5.8 million people affected by hypertension [8–10]. With the rate at which hypertension is accelerating due to such risks, it becomes a public health emergency worldwide. As observed in developing countries, the projection is that, by year 2025, there will be an increase of 80% in the number of hypertensive individuals [11].

However, recognition of psychosocial risks and work-related stress is still in infancy stage in industrial sector of Malaysia. Over the years, the Department Of Occupational Safety and Health and Social Security Organization of Malaysia have reported only 4 industrial cases as “psychosocial problems” under “types of diseases” and three hundred forty-four as mental health cases by year 2015. It is noteworthy that the majority of the other occupational diseases like physical, chemical, and biological agents and many other environmental factors are still on priority in Malaysian industries [12].

Consequently, psychosocial risks and work-related stress have been widely acknowledged as the global issues [13] and have affected many oil and gas and petrochemical industry workers of developed countries [14–17]. Due to chronic biological simulations, fluctuations in BP have been as an underlying mechanism through which psychosocial risks and work-related stress lead to a state of hypertension and ultimately result in cardiovascular diseases [18, 19]. The responses to acute stress are well documented, but the process by which work-related stress and psychosocial risks contribute to the BP is not well understood [19, 20]. Many past studies have investigated and shown mixed findings: either elevated BP, decrease in BP, or no effects on BP in relation to psychosocial risks and work-related stressors [19, 21–23].

For developing countries, a comprehensive framework for monitoring psychosocial risks and work-related stress is needed especially for specific sectors [25]. This enforces us to develop a psychosocial work environment model which induces health-related outcomes after investigating the influence of psychosocial work environment factors on industrial workers psychological and physiological health.

## 3. Theoretical Background and Study Hypotheses

The job demands-resources (JD-R) theory takes an assumption of employees' healthiness and wellbeing as an equalization of positive (resources) and negative (demands) job characteristics. In JD-R theory, psychological and physiological effects of job demands lead to psychological and physical health problems like escalated blood pressure, depression, and heart diseases [26].

Bakker and Demerouti defined job demands as “those physical, social, or organizational aspects of job that require sustained physical or mental effort and are associated with certain physiological and psychological costs,” such as high work pressure and emotionally demanding situations. Job resources are “those physical, social, or organizational aspects of the job that may do on any of the following: be functional in achieving work goals; reduce job demands and the associated physiological and psychological costs; stimulate personal growth and development”; examples are autonomy, skill variety, support, performance feedback, and opportunities for growth [27]. Many previous studies have provided little insight into how links of perceptions of job demands and job resources are forged into JD-R model [28]. The number of empirical studies on burnout (stress) has increased rapidly, although a comprehensive theoretical framework explaining it is still lacking [27].

### 3.1. Prevalence of Stress

For International Labor Organization stress is harmful physical and emotional effect caused due to the imbalance between the perceived demands and resources and the ability of the individual to work out with such demands [4]. Although stress itself is not a disease but it is the first sign of problem, work-related stress can contribute to sleeping troubles, memory loss, diabetes, obesity, peptic ulcers, inflammatory bowel diseases, musculoskeletal disorders, and high blood pressure which further leads to the development of heart and cardiovascular diseases and cancer. It may alter immune functions which in turn facilitate the development of cancer. Taken together, these disorders are responsible for majority of diseases, disability, and medical care use in most industrialized countries and also have significant causes of death in developing countries.

**Figure 1:**
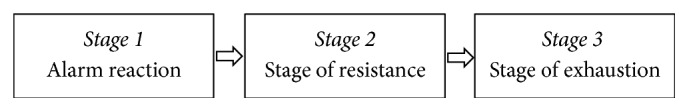
Three-stage stress syndrome or GAS [24].

The question here that arises is what actually stress is; “stress evolves when we must do something that we are not able and/or willing to do” [29]. In year 1965 Selye used the term stress as “a non-specific response of the body to any demand of change.” “Stress syndrome” or “General Adaption Syndrome (GAS)” simply shows that “stress” revolves around three stages shown in Figure 1 [24]. In stage 1, the defensive forces are mobilized and we start to lose control on our life. Workers act like as if they are in danger and their survival is at stake. At workplace when this happens, they usually are no longer being able to pay full attention to their work. These are “f-responses: either we become angry and suddenly start to fight, or we get scared and ready to flee, or we freeze as we startle” [29].

As our aim is to study the psychosocial work environment, therefore, the following psychosocial demands and resources were selected and hypothesized in lieu to job demands-resources theory:

 
H_1a_: quantitative demands significantly influence stress.
 
H_1b_: quantitative demands significantly influence mean arterial pressures (MAP).
 
H_2a_: emotional demands significantly influence stress.
 
H_2b_: emotional demands significantly influence mean arterial pressures (MAP).
 
H_3a_: work pace significantly influence stress.
 
H_3b_: work pace significantly influence mean arterial pressures (MAP).
 
H_4a_: work-family conflict significantly influence stress.
 
H_4b_: work-family conflict significantly influence mean arterial pressures (MAP).
 
H_5a_: role clarity significantly influence stress.
 
H_5b_: role clarity significantly influence mean arterial pressures (MAP).
 
H_6a_: job insecurity significantly influence stress.
 
H_6b_: job insecurity significantly influence mean arterial pressures (MAP).

## 4. Methodology

### 4.1. Participants and Procedure

The selected participants were the technical workers (operational, maintenance, production, etc.) working in the petrochemical industries of Malaysia. All the ethical protocols of the study have been fulfilled and well explained to the participants before the questionnaire distribution and repeated at the same time of measuring their blood pressure (BP). Participants were asked to come in room and relax themselves at least 30 minutes before giving their BP measurement. BP measurement of each participant was taken twice from left and right arm with 10 minutes of gap. Participants were advised that neither they nor the observer (member from research team) should talk to each other before and during the BP measurement. They have been further advised to take back support of the chair, with legs uncrossed, and that arm position at the time of measurement should be at the heart level.

Participants were asked to sign consent form before filling questionnaire. Two enumerators were hired during data collection on special services to help the respondents in understanding the translated items easily. The current study was conducted in Malaysia in which Bahasa Melayu (BM) is used as a national language; therefore all study variables were translated into BM from English using backtranslation technique [30]. Forward-then-back translation procedure was completed in multiple steps. Translation and backtranslation of internationally recognized base questionnaire into BM were carried out with the help of two certified translators located in Kuala Lumpur, Malaysia. In the first step English version has been translated into BM by one certified translator and, in the second step, the backtranslation from BM to English was done by another certified translator. In order to keep the originality and authenticity of both translations, the two translators who worked independently and unknown to each other were selected. To ensure each item's contents were cross-linguistically comparable and generated same meaning, both translated languages were incorporated in the single questionnaire.

**Figure 2:**
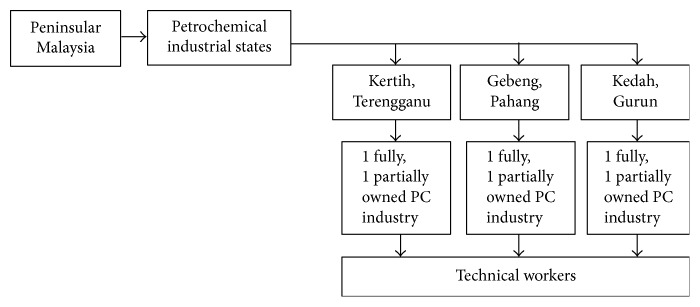
Multistage sampling for petrochemical industries of Malaysia.

In total 340 questionnaires were distributed by the research team in three different industrial zones of Malaysia using multistage sampling procedure as mentioned in Figure 2. Out of which 277 (81.47%) were usable while the remaining 63 (18.53%) were discarded on the basis of individuals who smoked, were on medication, used oral contraceptives, were pregnant, or had any other type of illness. The data was also normalized after removing outliers for further analysis.

### 4.2. Measures

Psychosocial factors were determined with the items and scales derived from second version of Copenhagen Psychosocial Questionnaire (COPSOQ II) [31]. “COPSOQ II is a tool for creating theoretical insight, an eye-opener for employees and employers,… a way to give legitimacy to the field of psychosocial factors at work…” [32].* Quantitative demands* were measured by 4 items' scale consisting of items like “do you have enough time for your work tasks?” (Adakah anda mempunyai cukup masa untuk menyiapkan tugasan kerja?).* Emotional demands* were measured by 4 items' scale consisting of items like “does your work put you in emotionally disturbing situation?” (Adakah kerja anda menyebabkan anda menghadapi situasi gangguan emosi?).* Work pace* was measured by 4 items' scale consisting of items like “do you have to maintain the high pace of work throughout the day?” (Adakah anda perlu mengekalkan kadar kerja yang cepat sepanjang hari?).* Work-Family Conflict* was measured by 4 items' scales consisting of items like “do you feel that your work drains so much of your energy that it has a negative effect on your personal life?” (Adakah anda berasa yang pekerjaan anda menghabiskan begitu banyak tenaga sehingga ia mempunyai kesan negatif ke atas kehidupan peribadi?).* Role clarity* was measured by 3 items' scale consisting of items like “does your work have clear objectives?” (Adakah kerja yang anda lakukan mempunyai objektif yang jelas?).* Job Insecurity* was measured by 4 items' scale consisting of items like “are you worried about new technology making you redundant?” (Adakah anda risau tentang Teknologi baru membuat anda tidak diperlukan?).* Stress* was measured by 4 items' scale consisting of items like “how often have you been stressed?” (Berapa kerapkah anda tertekan?).

All psychosocial factors in this study were measured with a five-point Likert scale (always, often, sometimes, seldom, and never/hardly ever).* Blood pressure* was measured as per practice guidelines of the European Society of Hypertension [33, 34]. We have used both psychological and physiological measures to overcome the issue of subjective measures (by using questionnaires only) and objective assessments (observational approaches or biological measures only) as highlighted in [35]. The mean arterial pressure* (MAP)* is defined as an average blood pressure in an individual during a single cardiac cycle as shown in the following equation:

(1) $$\begin{array}{c} MAP=SBP+\frac{2\left(DBP\right)}{3}, \end{array}$$

where SBP is the systolic blood pressure and DBP is the diastolic blood pressure. The unit for MAP measurement is mm Hg. MAP is used to approximate the pressure gradient (Δ*P*) of the subjects and includes the effect of systolic and diastolic pressure.

## 5. Results

### 5.1. Statistical Analysis

The second-generation technique Structural Equation Modeling allows researchers particularly in health to examine and analyze the complex and causal relationships in explaining the development of the phenomena such as diseases and health behaviors. The biggest advantage of using SEM in epidemiology studies over first-generation research techniques such as multiple regressions is that it is more powerful and has the ability to manage measurement error and the path coefficients are measured simultaneously [36]. Therefore, SEM methods as implemented by Analysis of Moment Structures (AMOS) were used for data analysis. The procedure suggested in [37] was employed to test the measurement model and the structural model.

#### 5.1.1. Measurement Model

**Figure 3:**
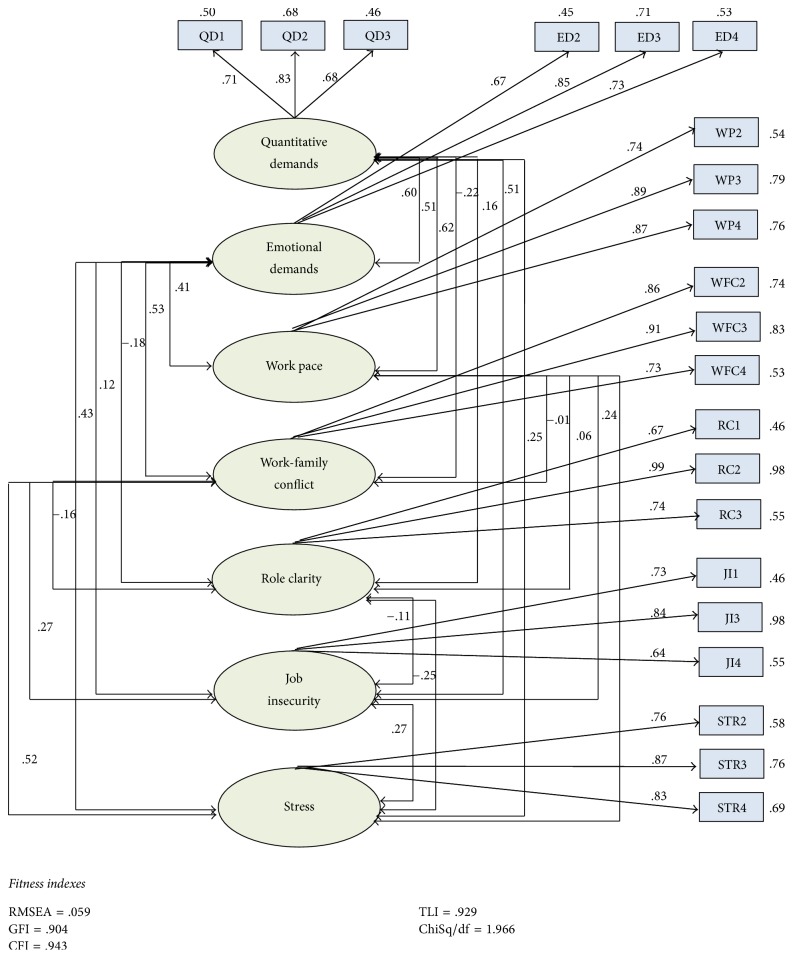
The measurement model with model fit results.

*(1) Model Fit, Convergent Validity, and Constructs Reliability*. The measurement model was analyzed using Maximum Likelihood Estimation (MLE) technique. Seven of the variables were covaried in a model to perform confirmatory factor analysis (CFA) to find out the model fit, reliability, and validity of the instruments. MAP was measured on ratio scale with single item; therefore, it was not part of measurement model. In order to achieve the validity of the model, six items were deleted.  Figure 3 shows the variables that are part of this study along with the overall measurement model fit indices.

The measurement model adequately achieved the model fit criteria as all the goodness-of-fit indices fall under the critical values. That is, *χ*^2^/df = 1.966 < 5.0, GFI = 0.904, AGFI = 0.868, CFI = 0.943, TLI = 0.929, and RMSEA = 0.059 < 0.50. Moreover, the residual covariances were less than 1.0 demonstrating that the model can predict the variance covariance matrix.

Following the model fit, the model was tested for constructs validity and composite reliability.  Table 1 shows the achieved construct and discriminant validity of study variables. The model achieved convergent validity if factor loadings of each indicator are greater than 0.6 and the average variance extracted (AVE) of all constructs is greater than 0.5 [38, 39].

The AVE for all constructs is greater than 0.5 and CR is greater than 0.7. Thus, the model achieved convergent validity [39]. Discriminant validity was established using the recommended techniques in [38]. The model has successfully achieved discriminate validity as square root of AVE between any two constructs is greater than their interconstruct correlations. Finally, the data was normalized and free from common method bias.

#### 5.1.2. The Structural Model

**Figure 4:**
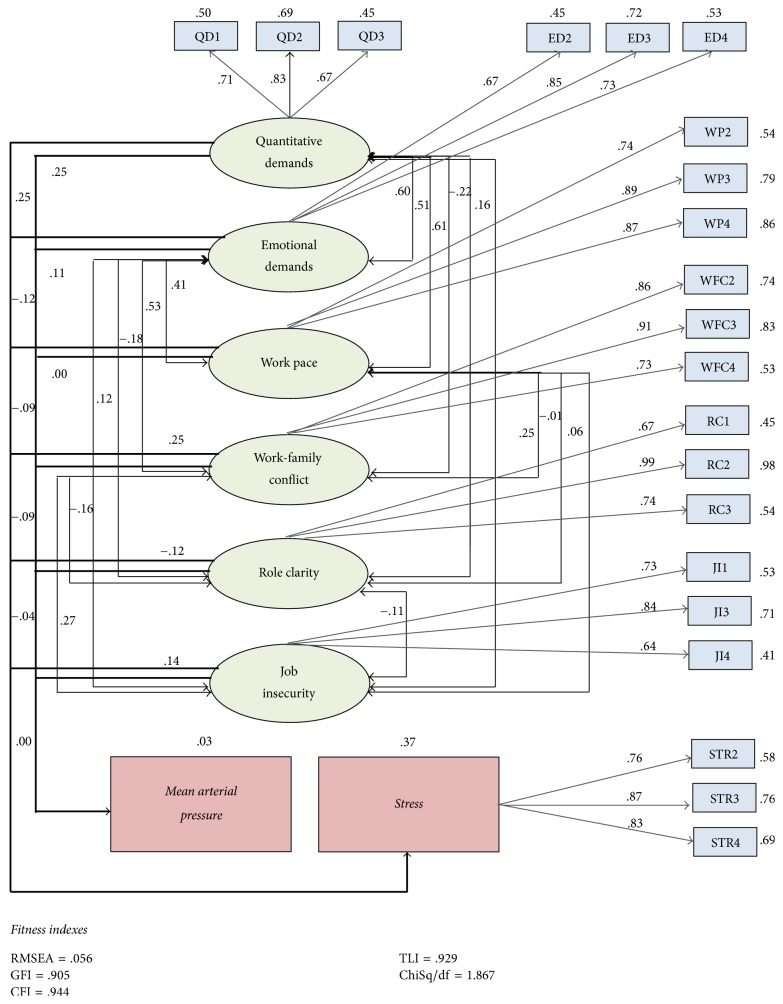
The structural model with model fit results.

After meeting measurement model criteria, structural model was estimated to test the proposed hypotheses. The goodness-of-fit statistics showed structural model adequately fits the data; that is, *χ*^2^/df = 1.867; GFI = 0.905; AGFI = 0.868; CFI = 0.944; TLI = 0.929; and RMSEA = 0.056. The established relationships in the model and goodness-of-fit indices are shown in Figure 4.

Stress is positively influenced by quantitative demands (*β* = 0.188, *t* = 2.311, and *p* = 0.021), work-family conflicts (*β* = 0.201, *t* = 2.848, and *p* = 0.004), and job insecurity (*β* = 0.095, *t* = 2.142, and *p* = 0.032) and is negatively influenced by role clarity (*β* = −0.102, *t* = −2.04, and *p* = 0.041) and, therefore, supported H_1a_, H_4a_, H_5a_, and H_6a_ respectively. Nonetheless, in contrast to the predictions, work pace negatively influences stress (*β* = −0.002, *t* = −0.028 and *p* = 0.978) and interestingly emotional demands have no significance. Thus H_2a_ and H_3a_ are insignificant and are not supported.

The next step was to test predictors with mean arterial pressure (MAP). Findings showed mean arterial pressure is positively influenced by only quantitative demands (*β* = 3.714; *t* = 2.078; *p* = 0.038), whereas other psychosocial factors have no influence on it. Thus, a hypothesis H_1b_ is supported but H_2b_, H_3b_, H_4b_, H_5b_, and H_6b_ are not supported. The details of overall supported and not supported relationships between dependent and independent variables are shown in Table 2 of this study.

## 6. Discussion

Profound changes in the ways in which work is organized and carried out have taken place over the last many years, particularly in the western world and more recently in the rapidly industrializing nations of Asia [40]. During the past 30 years, the number of studies related to psychosocial work environment on employee health has increased steadily with the amount and pace of work being an important concern for wellbeing and performance at work [41, 42]. As the pace of competition increased and a truly global marketplace developed, occupational stress and its consequences have greatly increased too. Increased work hours, increased pressure, increased insecurity, and many other organizational stressors were shown to have immediate and long-term deleterious consequences for both individuals and organizations.

Aim of this study was to investigate the influence of psychosocial work environment factors on health of the workers working in the petrochemical industries of Malaysia. We predict stress and mean arterial pressure as the health measuring variables in relation to job demand variables such as quantitative demands, work pace, and emotional demands besides work-family conflicts and job insecurity and job resources such as role clarity to act as a buffer in minimizing the effects of job demands. Main findings indicated both job demands and job resources predicted health of workers in line with the expectations of job demands-resources JD-R theory.

Previous studies have confirmed quantitative job demands such as workload, time pressure, and unclear roles in combination with lack of resources such as control over job, rewards, and social support as the main sources of stress. Dollard et al. concluded that results were consistent with JD-R model; that is, high job demands when combined with low job resources were associated with adverse health outcomes [28].

We found significant impact of quantitative demands, work-family conflict, and job insecurity on workers stress in the petrochemical industries of Malaysia. One of the arguments is that employers in developing countries in comparison to developed countries are more focused on the production and quantity of the product rather than the safety and health of their workers [43]. This results in an intense work environment for the workers where they started to lose control on their work which significantly affects individuals' health. We argued that reducing stressful work environment, having impartial workplace, and being nice to others are ethical imperatives in epidemiological studies but empirical evidence of using role clarity as the only intervention has not supported our hypothesis. This shows that other psychosocial interventions such as rewards, influence at work, sense of community at work, and social support will likely act as buffering solution for the manufacturing industry workers in general and in particular to the petrochemical industry workers. This will not only help in improving the health but also help in improving the behavioral responses, psychological risk profiles, and mental disorders of workers. In Malaysia, Department of Occupational Health and Safety has already published the guidelines for prevention of stress at workplace and highlighted the mental disorders issues in their national list of occupational diseases. Also, an integral part of the economic sustainability and organizational development relates to innovative approaches with more focus on workers' health, safety, and wellbeing.

Insignificance of demands is due to the fact that we have distinguished between quantitative demands (i.e., high workload) and work pace (i.e., high speed) as per suggestions and guidelines provided [44]. Therefore, at one point of time workers may feel that they have high quantitative demands rather than high work pace and at another point of time they have to maintain high work pace instead of high quantitative demands. Hansen et al. posit an argument that self-selection of tasks at times creates different tiredness level in male and female workers which basically depends upon the psychological and physiological perceptions. For example, females who choose to work at high pace may be particularly more strong and healthy in comparison to male counterparts, due to the perception they made for that task [45]. Our findings are consistent with the findings in [46] where authors found weak association of work pace with the psychiatric disturbance and it was not significantly associated with the wellbeing.

These psychosocial risks can further be synchronized on the basis of “task analyses” which the technical workers perform. Task analysis normally evaluates human-human and human-machine interaction. The purpose of psychological task analysis is to lead to more efficient and effective integration of human factors into system designs and operations with the help of task design or redesign to optimize human performance, avoid health-related negative outcomes, and increase safety. If assigned tasks got psychologically irrelevant characteristics then there are high chances of fluctuations in BP, which are supported by results. These tasks are characterized on the basis of four different behavioral approaches [42]:

- Behavior Description Approach
- Behavior Requirement Approach
- Ability Requirement Approach
- Task Characteristics Approach

*Behavior Description Approach.* Behavior Description Approach is the approach where focus is on the actual behavior of the workers at the time of conducting a task in certain conditions. Any psychological distresses such as excessive demands at work may affect the workers attention at the time of operational activity performed at plant. The study results showed that in petrochemical industries too high job demands experienced by the workers result in an impaired health which ultimately leads to poor job performance by them at workplace. Moreover, hazardous industrial jobs such as petrochemical industrial environment are inherently risky that bring along the psychological effects and also comprise the cognitive, emotional, and physical dimensions which is in agreement with the job demands-resources (JD-R) theory and demand support control model (DSCM).

Job strain in DCSM indicates that job demands can be detrimental to health by putting both psychological and physiological load on the workers-A Strain Response [47]. These loads geared the workers towards stress where there are chances to have elevated level of BP and heart rate along with depression and certain heart diseases. If workers cannot cope up with job demands then the workers will not recover their mental and physical energy and the stress response will be prolonged [47]. The results are in concordance with earlier findings which showed that when quantitative demands and other work-related stress factors are high, then there is an elevation of sympathetic nervous system activity during the work time, leading to increase in blood pressure [18, 48].

*Behavior Requirement Approach*. It is the approach where focus is on the actual behavior that worker should need to display for the safe completion of the task. If the focus of the worker is not entirely on the tasks to be performed, showing lack of dedication and concentration, then there are high chances of unsafe acts that lead to health and wellbeing issues. Psychosocial risks are associated with distracting workers psychologically and change actual behaviors of the workers to a large extent. This shows that occupational conditions related to the social environment discern an immediate impact on biological functioning and have cumulative long-term effects on the petrochemical workers' health. Similarly, the tasks performed by the workers in the operational activities at plant are associated with certain emotions. Some tasks are pleasant to do, some are unpleasant, some arouse interest, and some are boring. For instance, production of petrochemical products or the maintenance of any equipment at plant may have the repetitive process over a period of time and could create behavioral distraction at any point that results in injuries and other health-related outcomes. The JD-R theory suggests that stressors lead to strain reactions and further strengthen results by providing a lens on relationship where psychosocial risks cause stress.

*Ability Requirements Approach*. Here the tasks are analyzed according to the abilities, skills, and personal characteristics of the workers. If the tasks are unequally distributed amongst the workers, that is, a mismatch between the knowledge and ability possessed by the workers and their personal characteristics are different with the assigned jobs, then there are maximum chances of job strain which ultimately affects the health and wellbeing. Therefore, it is important to maintain the person-environment fit. 

*Task Characteristics Approach*. Task characteristics approach analyzes objective characteristics of the task rather than behavioral approaches, that is, actual requirement of the task that should be displayed. Such type of tasks in the industry varies in lieu to the production activities. For instance, too much demand at work with limited control at work exerts added pressure over the technical workers which leads to blood pressure fluctuations. The inherent hazardous nature of industry yielded stressful job and may promote such behaviors which are hazardous for workers' health.

## 7. Conclusion

Malaysian government has always adopted a balanced development approach that gives equal emphasis to both economic growth and the wellbeing of the ordinary people (rakyat in Bahasa Melayu). The concept of 1Malaysia is founded upon the aspiration of building a harmonious, progressive, and united Malaysian society, based on the underlying principle of “People First, Performance Now.”

The study provides an integrative theoretical framework and empirical evidence to discuss work-related stress factors and health biomarker. Findings suggest that the development of health symptoms is determined by specific constellation of working conditions. When demands are high, workers experienced increased health issue and when resources are there then workers faced buffering effect of clear roles towards demands.

From the managerial point of view, it is important to control the level of workplace demands and also increase the resources. Quantitative demands were found to be the only demands that were affecting our endogenous variables that are stress and MAP. Therefore, it is significantly important for petrochemical managers to carefully monitor the amount of workload, work pressure, and working hours of workers in order to make them more effective for the organization. We have also found that role clarity as job resource in the presence of many demand factors at work has significantly helped to decrease the stress (negative relationship).

One important question is why still there is so little that is being done in developing countries in the area of occupational health in general and psychosocial risks in particular and Malaysia is no exception to it. Some experts claim that inadequacy of knowledge of psychosocial risks impedes addressing of occupational health-related outcomes, partly due to the fact that other health issues due to physical conditions are the priority. Another general issue pertains to the fact that occupational diseases emanating from some physical risks are not included in the definition of easily preventable diseases; neither are any psychosocial risks that are affecting workers' health. Future work may examine the widening of job demands and resources variables to fully understand the psychosocial work environment and also how biomarkers can be used with psychosocial risks as a diagnosing tool to identify the impact on the health of the workers. This may help mainstream policy makers in proper recognition and regulation of these health hazards. The study has highlighted those psychosocial risks which are widespread in affluent and transitional economies to get a better idea of psychosocial work environment model.

## Figures and Tables

**Table 1:** Construct and discriminant validity.

Measurement model	CR	AVE	WFC	RC	WP	JI	STR	QD	ED
Work-family conflict	0.87	0.70	**0.83**						
Role clarity	0.85	0.65	−0.16	**0.81**					
Work pace	0.87	0.69	0.25	−0.01	**0.83**				
Job insecurity	0.78	0.54	0.27	−0.11	0.06	**0.74**			
Stress	0.86	0.67	0.52	−0.25	0.24	0.27	**0.82**		
Quantitative demands	0.78	0.54	0.62	−0.22	0.51	0.16	0.51	**0.73**	
Emotional demands	0.79	0.56	0.53	−0.18	0.41	0.12	0.43	0.60	**0.75**

*Note*. WFC = Work-family conflict; RC = role clarity; WP = work pace; JI = job insecurity; STR = stress; QD = quantitative demands; ED = emotional demands; CR = composite reliability; AVE = average variance extracted; bold values = square root of AVE's (values are greater than interconstruct correlations).

**Table 2:** Structural model estimates.

H	IV	Path	DV	*β*	SE	*t*	*p*	Results	Remarks
H_1a_	QD	→	Stress	0.217	0.094	2.307	0.021	Significant	Supported
H_2a_	ED	→	Stress	0.096	0.073	1.318	0.187	Insignificant	Not supported
H_3a_	WP	→	Stress	−0.002	0.06	−0.028	0.978	Insignificant	Not supported
H_4a_	WFC	→	Stress	0.201	0.071	2.848	0.004	Significant	Supported
H_5a_	RC	→	Stress	−0.102	0.05	−2.04	0.041	Significant	Supported
H_6a_	JI	→	Stress	0.095	0.044	2.142	0.032	Significant	Supported
H_1b_	QD	→	MAP	3.714	1.788	2.078	0.038	Significant	Supported
H_2b_	ED	→	MAP	−1.801	1.392	−1.294	0.196	Insignificant	Not supported
H_3b_	WP	→	MAP	−1.207	1.132	−1.066	0.286	Insignificant	Not supported
H_4b_	WFC	→	MAP	−1.262	1.335	−0.946	0.344	Insignificant	Not supported
H_5b_	RC	→	MAP	−0.662	0.943	−0.702	0.483	Insignificant	Not supported
H_6b_	JI	→	MAP	0.042	0.835	0.05	0.96	Insignificant	Not supported

*Note.* WFC = Work-family conflict; RC = role clarity; WP = work pace; JI = job insecurity; ST = stress; QD = quantitative demands; ED = emotional demands; H = hypotheses; IV = independent variable; DV = dependent variable.
